# Experimental Visualization of Labyrinthine Structure with Optical Coherence Tomography

**Published:** 2017-01

**Authors:** Saleh Mohebbi, Marjan Mirsalehi, Lüder-Alexander Kahrs, Tobias Ortmaier, Thomas Lenarz, Omid Majdani

**Affiliations:** 1 *Brain and Spinal cord Injury Research Center, Neuroscience Institute, Tehran University of Medical Science, Tehran, Iran.*; 2 *Institute of Mechatronic Systems, Leibniz University Hannover, Hannover, Germany.*; 3 *Department of Otorhinolaryngology, Hannover Medical School, Hannover, Germany.*

**Keywords:** Cochlea, Decalcification, Optical Coherence Tomography, Labyrinth

## Abstract

**Introduction:**

Visualization of inner ear structures is a valuable strategy for researchers and clinicians working on hearing pathologies. Optical coherence tomography (OCT) is a high-resolution imaging technology which may be used for the visualization of tissues. In this experimental study we aimed to evaluate inner ear anatomy in well-prepared human labyrinthine bones.

**Materials and Methods:**

Three fresh human explanted temporal bones were trimmed, chemically decalcified with ethylenediaminetetraacetic acid (EDTA), and mechanically drilled under visual control using OCT in order to reveal the remaining bone shell. After confirming decalcification with a computed tomography (CT) scan, the samples were scanned with OCT in different views. The oval window, round window, and remnant part of internal auditory canal and cochlear turn were investigated.

**Results:**

Preparation of the labyrinthine bone and visualization under OCT guidance was successfully performed to a remaining bony layer of 300µm thickness. OCT images of the specimen allowed a detailed view of the intra-cochlear anatomy.

**Conclusion:**

OCT is applicable in the well-prepared human inner ear and allows visualization of soft tissue parts.

## Introduction

Inner ear structures – which are embedded in dense temporal bone – are the main organs for hearing and balance. Because of the increasing trend toward preserving residual hearing, visualization of the anatomical details of the inner ear is of considerable interest. Increasing anatomical knowledge helps improve our understanding of hearing pathology and interventions, while hearing preservation techniques rely on the exact position of membranous structures and cochlear implant electrodes. For several decades, different imaging systems have been used for histologic and anatomic scanning, including computed tomography (CT) and magnetic resonance imaging (MRI) (1,2). Because of their higher resolution, micro-computed tomography (µCT) and scanning laser optical tomography (SLOT) provide an improved option for scanning and highlighting anatomical details (3,4).

Optical coherence tomography (OCT), a high-resolution imaging technology which was introduced in medicine more than 30 years ago (5), is an attractive technique for the visualization of surfaces and subsurfaces. OCT is a B-mode modality based on near infra-red light.

An OCT system delivers near infra-red light and collects the reflected light from the subject (referred to as A mode). Using a scanning mirror it is possible to build two- and three-dimensional (2D,3D) images (6). Tissue density is an essential factor affecting the depth of light penetrance and imaging. In soft tissue, light can reach 1–3 mm inside the structure (7); however penetration depth is limited in the bone. OCT resolution is close to that of confocal microscopy and better than that of ultrasonography or MRI (8). OCT has previously been used in studies of the middle ear study and surgical techniques (9-11). Using OCT in image-guided surgery is an interesting proposition in ear surgery, including cochlear implantation (12). As the human inner ear structure is not easily accessible for OCT scanning, many researchers have previously focused on animal cochlea.

In this experimental study we aimed to scan well-prepared labyrinthine bone using OCT, and assess the feasibility of this technique for further areas such as microanatomy studies, inner ear disorders, and implantation.

## Materials and Methods

Three fresh, explanted human temporal bones were used in this study. The samples were trimmed into a cubic form (approximately 2×2×2 cm3). After fixing the samples with formol 4% overnight in the freezer (−18°C), decalcification proceeded with ethylenedia- minetetraacetic acid (EDTA 20%), according to a previously established protocol (13).

 The OCT scanner (Thorlabs Swept Source OCS1300SS) was used for guidance during mechanical decalcification by use of a drill. The samples were agitated on the orbital shaker and mechanically milled under OCT visualization. Milling was frequently repeated for removal of the softened bones. The EDTA medium was changed intermittently after each treatment. The process was repeated until the subsurface became visible and the samples became translucent on CT images. The entire anatomy of the decalcified samples was assessed using OCT. Different parameters and values were measured with this method using OCT software.

## Results

The decalcification process took less than 8 hours to complete. The most time-consuming part was drilling the vestibular side of the samples, because of its complexity in shape. Preserving the membranous part of the labyrinth was critical to guarantee the intactness of the inner structures. 

**Fig 1:**
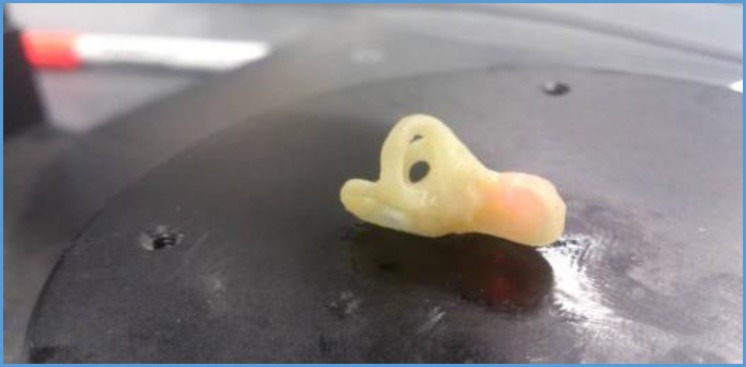
OCT system and a decalcified cochlea

The OCT system used, and an example of a decalcified cochlea, are shown in Figure 1. All recorded measurements are included in the final line of the screenshots.

**Fig 2:**
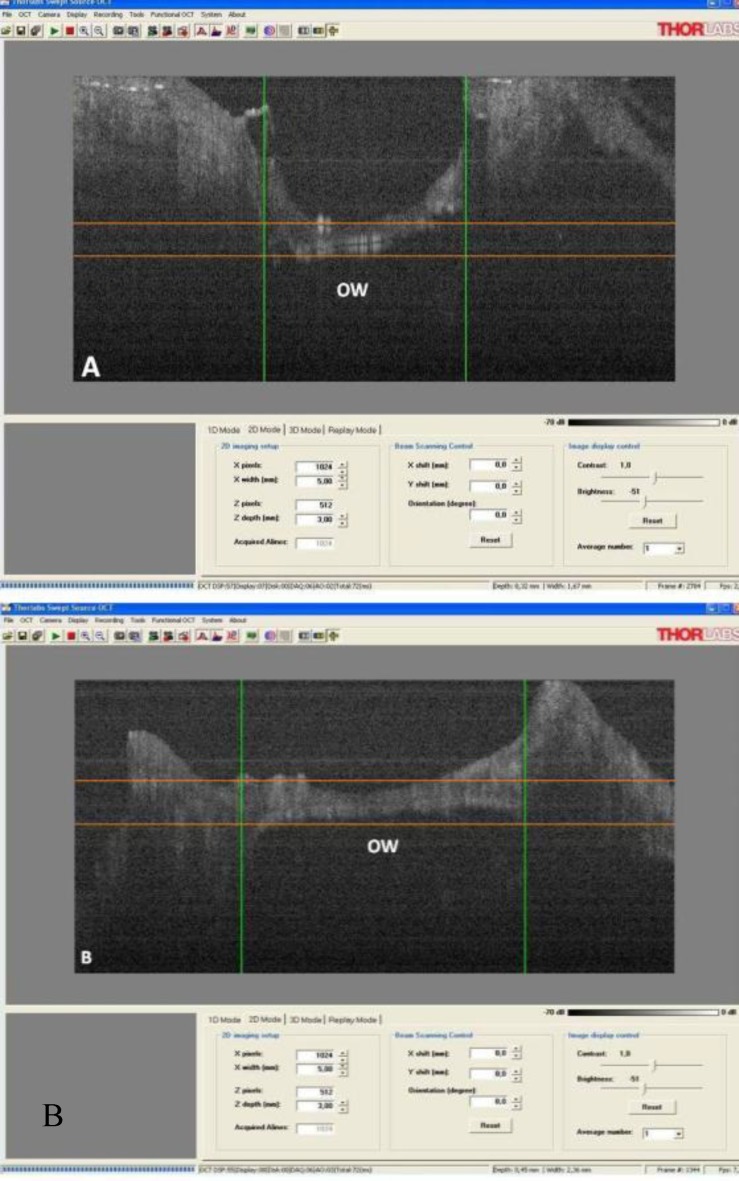
Visualization and measuring of a foot-plate in the oval window, A) Vertical view, B) Horizontal view

Visualization and measuring of the foot-plate (FP) in the oval window is demonstrated in Figure 2. The average vertical and horizontal diameters were 1.67mm and 2.36mm, respectively. These measures were extracted from software, which appear in lasat line of screenshot. The well-preserved FP in sample number two was thicker on the periphery and thinner toward middle part, with an anterior trend. The thinner part measured 0.28mm and thicker posterior part measured 0.70mm. The thickness of the anterior part was 0.45mm. However, some parts of the FP had resorbed during the procedure.

**Fig 3:**
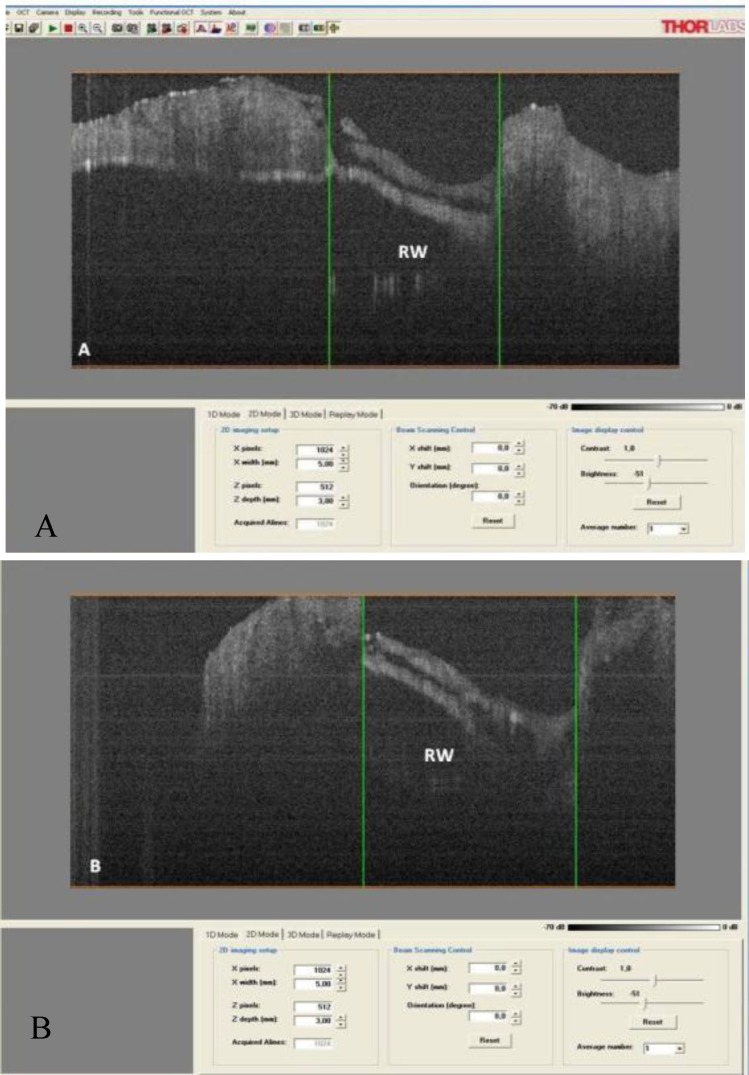
Round window (RW) visualized after removing the RW niche. A) Vertical view, B) Horizontal view

The round window (RW) was visualized after removing the RW niche (Fig.3).

**Fig 4:**
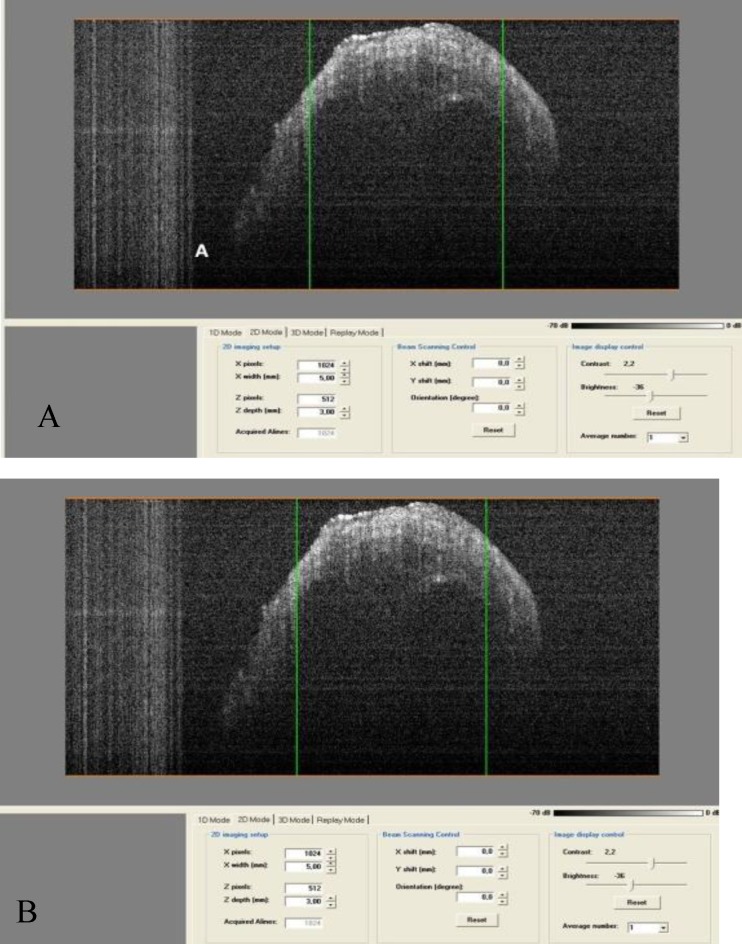
Scan of the first turn of the cochlea.

**Fig 5:**
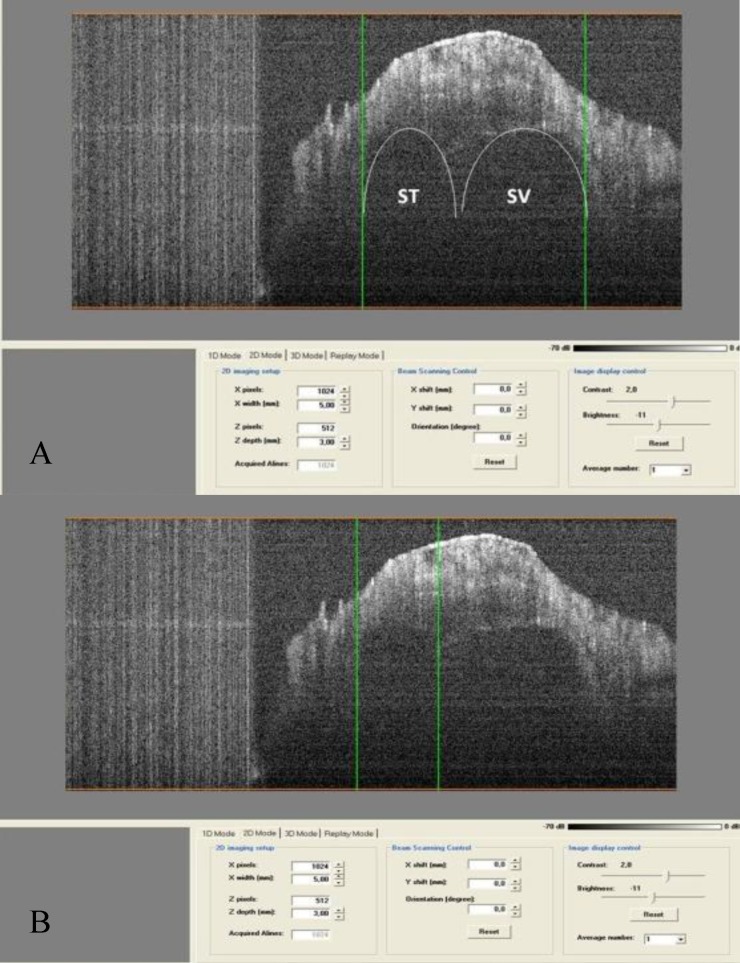
A) End of first turn in lateral view, B) Scala tympani

The image showed the lopsided position of the RW membrane in the anterior and superior part. The vertical and horizontal diameters of the RW were 1.76mm and 1.40mm, respectively. However the membrane was oblique. Because of its spiral shape, scanning of the cochlea requires rotation of the sample. All turns of the cochlea were scanned. Figure 4 shows a scan of the first turn of the cochlea, as this is the most important turn with respect to pathology and intervention. The width of this turn was initially 1.60mm, and the width of the scala tympani (ST) was 0.65mm. The basilar membrane was visualized in a lateral projection with a width of approximately 0.30mm. In the final part of first turn, the width was 1.83mm and the ST diameter was 0.67mm (Fig. 5). 

A) Beginning of first turn, B) Scala tympani

**Fig 6:**
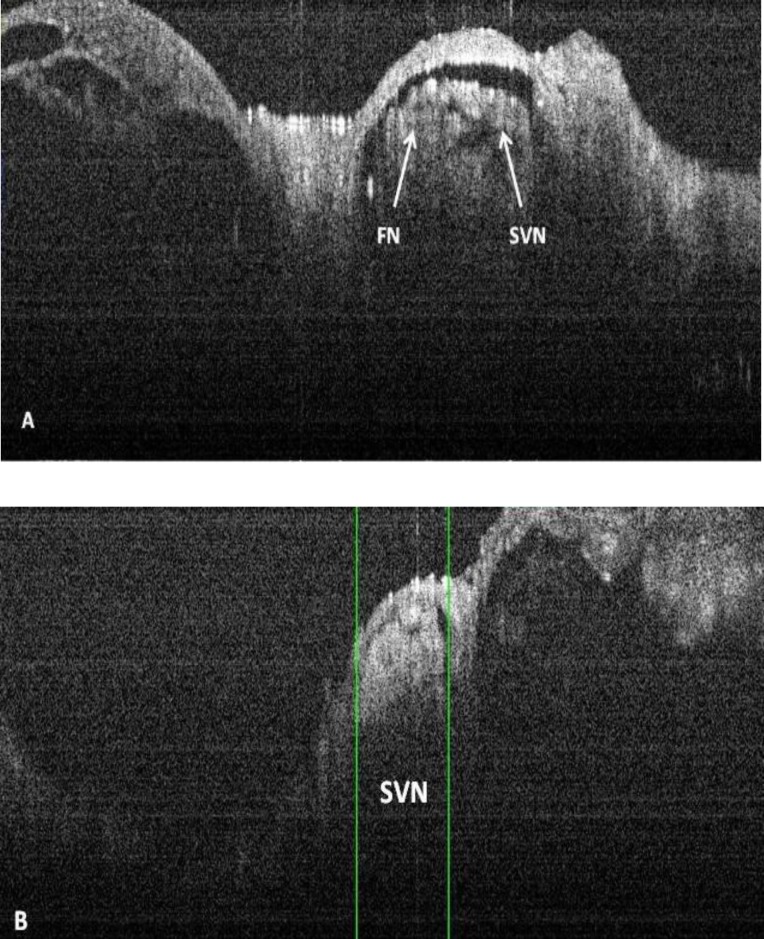
A) facial nerve and superior vestibular nerve (SVN) B) SVN before entrance to vestibular organ

The remaining part of internal auditory canal was also scanned. Using OCT, we were able to sharply visualize a defining line between the facial nerve and the superior vestibular nerve (SVN). The width of the SVN immediately before its entrance to vestibular organ was 0.6mm (Fig.6).

## Discussion

The scanned images obtained from different parts of inner ear show that this process is feasible. The high-resolution images were highly suitable for outlining the different structures and for the precise measurement of the desired parts.

OCT is currently widely used in research studies of the ear and inner ear. Due to the thickness of the otic capsule bone in humans, most studies are performed in animals (14-16). One of the most important aspects of inner ear visualization is increasing the knowledge of the detailed anatomy of the inner ear, which is important for studying hearing diseases and interventions such as cochlear implantation. It has also become more important in hearing preservation procedures. With an image resolution close to 10µm, OCT is very attractive for use in precise procedures such cochlear implantation (17). Furthermore, OCT has recently been used for imaging during cochleostomy (13,14) and is also included in laser systems in experimental cochlear implant surgery (18).

Wong et al. described the feasibility of recording an OCT image in rats (15). They identified the scala vestibuli, scala media, ST, modiolus, spiral ligament, and several turns of the cochlea. Similarly, Gao et al. reported a study of mouse cochlea imaging using OCT (16). In the Gao study, quantitative measurements confirmed the ability to detect critical changes relevant to hearing that were limited in other imaging modalities. Tona et al. scanned mouse cochlea in vivo (17), and applied the technique in the detection of endolymphatic hydrops. In most sites of decalcified cochlea, the remaining bone thickness was approximately 300–400μm. Greater decalcification may lead to clearer images. Using OCT for the detection of a wave in the tectorial membrane and basilar membrane is another potential application of this system (19). Iyer et al. used μOCT for microanatomical studies of the inner ear of the guinea pig and investigated its potential utility in otology research (20). Finally, Ramamoorthy et al. used OCT to assess sound processing in a non-opened otic capsule (21). These studies show the real potential of OCT in improving our knowledge of the inner ear and the potential of this technology in human inner ear proceduers. Future work will assess the microanatomy of the ear after greater preparation, and will also assess the ability of OCT to detect the electrode position of a cochlear implant in relation to other structures. Using OCT as a tool to visualize the human inner ear is a valuable step toward future applications in terms of guiding procedures and assessing disease pathophysiology.

## Conclusion

Using OCT allows detection of a well-prepared inner ear structure at a high resolution. Furthermore, OCT produces images of sufficient resolution to allow quantitative measurements of labyrinthine structures without tissue injury.
